# Evaluation of therapeutic efficacy, safety and economy of ERCP and LTCBDE in the treatment of common bile duct stones

**DOI:** 10.3389/fphys.2022.949452

**Published:** 2022-08-25

**Authors:** Renjie Zhang, Jialin Liu, Huizhen Li, Qingteng Zeng, Shenfeng Wu, Hengyu Tian

**Affiliations:** ^1^ Department of Hepatobiliary Surgery, Shenzhen Traditional Chinese Medicine Hospital/The Fourth Clinical Medical College of Guangzhou University of Chinese Medicine, Shenzhen, China; ^2^ The Fourth Clinical Medical College of Guangzhou University of Chinese Medicine, Shenzhen, China

**Keywords:** choledocholithiasis, ERCP, LTCBDE, efficacy, safety, cost

## Abstract

**Objectives:** This study further compared the endoscopic retrograde cholangiopancreatography (ERCP) and laparoscopic transcystic common bile duct exploration (LTCBDE) approaches in the treatment of common bile duct stones (CBDS) from the perspective of efficacy, safety and economy.

**Methods:** The therapeutic efficacy and safety of ERCP and LTCBDE approaches were retrospectively compared. Cost-effectiveness analysis of clinical economics was performed to analyze and evaluate the two approaches.

**Results:** There was no significant difference in the success rate of surgery and bile stone residue between ERCP and LTCBDE group. The incidence of postoperative complications in ERCP group was significantly higher than that in the LTCBDE group; while the incidence of pancreatitis in the ERCP group was significantly higher than that in the LTCBDE group. There was no significant difference in biliary infection, bile leakage and sepsis between ERCP and LTCBDE groups. In terms of cost, the costs of surgery and nursing were significantly lower, the costs of treatment and sanitary materials were significantly higher in the ERCP group than that in the LTCBDE group. There was no significant difference in the costs of medical examination, laboratory test, medicine cost and total cost between ERCP group and LTCBDE group. The total length of hospital stay, length of hospital stay before surgery and duration of surgery in the ERCP group were significantly lower than that in the LTCBDE group; there was no significant difference in length of hospital stay after surgery between the ERCP and LTCBDE group. The cost-effectiveness ratio of ERCP group was 34171.25, and the cost-effectiveness of LTCBDE group was 34524.25. The incremental cost-effectiveness ratio (ICER) of the two groups was 51415.

**Conclusion:** ERCP and LTCBDE approaches had similar therapeutic efficacy in the treatment of CBDS. The safety of LTCBDE approach is superior to that of ERCP approach for the treatment of CBDS. ERCP approach is more economical in the treatment of CBDS than LTCBDE approach.

## 1 Introduction

Gallstones represent a common condition in the general population, 10% of which may have specific medical conditions, such as acute cholecystitis and biliary pain. Only 1–2% of affected people have serious complications (Gracie and Ransohoff, 1982; Friedman, 1993; Shaffer, 2006). The migration of stones into the common bile duct can obstruct the bile flow in the small intestine, resulting in pain, jaundice, and sometimes cholangitis, which largely contributes to the symptoms and major complications of affected people (Soltan et al., 2001; Williams et al., 2008). Primary choledocholithiasis refers to stones formed directly within the biliary tree. Primary stones are generally brown and composed mainly of calcium bilirubin; these stones are rare in Western populations and more common in Asia, but the exact etiology and overall prevalence remain unclear (Williams et al., 2017). Secondary choledocholithiasis refers to stones migrated from the gallbladder, and its stone composition parallels that of cholelithiasis with cholesterol as the most common type (Williams et al., 2017). The presence of common bile duct stones (CBDS) represents 5–15% of the total of cholecystectomies performed every year for cholelithiasis (Vilallonga et al., 2012). The management of CBDS represents a significant clinical problem. In symptomatic patients, the primary goal is to obtain complete clearance of the common bile duct and cholecystectomy; on the contrary, in asymptomatic patients, there is still no shared diagnostic and therapeutic path (Cianci and Restini, 2021). In the last 20 years, the development of new technologies has allowed new diagnostic and therapeutic scenarios with a consequent critical evaluation of management options. All these have led to a more cautious and patient-tailored preoperative workup based on the patient’s risk and ultimately to a multidisciplinary approach (Ong et al., 2005).

Choledocholithotomy was once the treatment of choice for choledocholithiasis. However, traditional laparotomy is more traumatic with more intraoperative bleeding, slower postoperative recovery, and a higher incidence of postoperative complications (Sanchez et al., 2010). In 1973, Kawai et al. carried out the first endoscopic sphincterotomy (EST) of the duodenal papilla (Kawai et al., 1974); in 1982, Staritz et al. reported the endoscopic papillary dilatation technique (EPBN); In 1991, Stoker et al. performed laparoscopic transcystic common bile duct exploration (LTCBDE) (Petelin, 2003), which avoided the disadvantages of traditional laparotomies (Kenny et al., 2014). At present, it is recommended that patients with choledocholithiasis be treated with minimally invasive surgery promptly after diagnosis to reduce iatrogenic trauma or complications caused by surgery based on expelling the stones (Baiu and Hawn, 2018). The endoscopic retrograde cholangiopancreatography (ERCP) and LTCBDE approaches have become two different minimally invasive treatments for choledocholithiasis (Guo et al., 2021). Their advantages of good curative effect, small trauma, quick recovery, and fewer complications have been recognized by the majority of medical workers (Guo et al., 2021). This study further compared the ERCP and LTCBDE approaches in the treatment of CBDS from the perspective of efficacy, safety and economy, to provide an essential reference for the majority of medical workers and patients in the choice of treatment methods for choledocholithiasis.

## 2 Materials and methods

### 2.1 Patient and public involvement

Patients or the public were not involved in the design, or conduct, or reporting, or dissemination plans of our research.

### 2.2 Patients

A single-center retrospective cohort study was performed by reviewing the electronic medical charts of 89 patients with choledocholithiasis admitted to the First Affiliated Hospital of Fujian Medical University from January 2015 to December 2016. Among these patients, 46 patients received ERCP as a treatment for bile stones (assigned as ERCP group); 43 patients received LTCBDE as a treatment for bile stones (assigned as LTCBDE group). This study was approved by the Ethics Committee of the First Affiliated Hospital of Fujian Medical University.

### 2.3 Inclusion and exclusion criteria

The inclusion criteria were as follows: 1) Patients were diagnosed with choledocholithiasis by ultrasound of the upper abdomen, computed tomography of the upper abdomen, percutaneous transhepatic cholangiography, magnetic resonance imaging of the upper abdomen, magnetic resonance cholangiopancreatography or ERCP; 2) All the patients had complete medical records. The exclusion criteria were as follows: 1) Undiagnosed cases of choledocholithiasis, or choledocholithiasis combined with hepatolithiasis; 2) Cases of biliary tract tumors found before or during surgery; 3) Common bile duct deformities or strictures; 4) Patients with heart, lung or kidney disease, or other serious underlying diseases who cannot tolerate surgery.

### 2.4 Surgical strategy

#### 2.4.1 Endoscopic retrograde cholangiopancreatography approach

After half an hour of routine intramuscular injection of diazepam and raceanisodamine, the patient was laid on the left side or prone position. The ERCP was performed via a duodenal endoscope in a standard manner by an endoscopist and his first assistant in our biliary department. Following deep cannulation, retrograde cholangiography, sphincterotomy or balloon expansion, the CBDS were extracted by a basket until no stones were confirmed by a repeat cholangiogram. Mechanical lithotripsy was used to retrieve the stones if needed. Serum amylase was detected 2 and 24 h postoperatively and subsequent laparoscopic cholecystectomy (LC) was performed within 3–5 days.

#### 2.4.2 Laparoscopic transcystic common bile duct exploration approach

The cystic duct close to the gallbladder was clipped and the distal cystic was reserved temporarily to access the common bile duct. The cystic duct was cut transversely at a distance of 1–2 cm to CBD, after which a catheter or balloon was used to dilate the cystic duct. If the diameter of the cystic duct <5 mm and >3 mm, a 3 mm choledochoscope was inserted through the incision to explore the CBD. If the diameter of cystic duct ≥5 mm, we used 5 mm choledochoscope. For patients with the diameter of stone size/cystic duct ≥1, we made a T-shaped incision at the confluence of the cystic duct and CBD and used electrohydraulic lithotripsy or biopsy forceps for stone fragmentation. A stone basket and saline irrigation were routinely used to retrieve the stones. After confirming that there was no retained stone, the cystic duct was ligated near the CBD by an absorbable clip or was sutured.

### 2.5 Therapeutic efficacy evaluation

The success of the surgery was defined as the removal of CBDS, laboratory test results being normal and the absence of postoperative clinical symptoms. Stone residue was defined as CBDS not being completely removed during the surgery, or postoperative detection of CBDS by T-tube choledochoscopy, ultrasound of upper abdomen, computed tomography of the upper abdomen, magnetic resonance imaging of upper abdomen or magnetic resonance cholangiopancreatography.

### 2.6 Safety evaluation of endoscopic retrograde cholangiopancreatography and laparoscopic transcystic common bile duct exploration

#### 2.6.1 Intraoperative injury

Cases of intra-abdomunal organ and tissue damage were found during surgery, post-operative imaging examination, post-operative endoscopy procedures, and during re-operation.

#### 2.6.2 Postoperative complications

Short-term complications include: 1) postoperative complications: the number of cases with postoperative complications; 2) biliary tract infection: postoperative clinical symptoms of cholangitis such as right upper quadrant pain, chills, fever; biochemical examinations showed increased inflammatory indexes; some patients had increased total bilirubin; 3) Pancreatitis: symptoms of abdominal pain of pancreatitis, blood amylase elevated to more than 3 times the normal value; 4) Bile leakage: abdominal drainage tube single-draining bile ≥100 ml/d or continuous 3 d bile outflow, no abdominal drainage tube, symptoms and signs of peritonitis confirmed bile by imaging examination and abdominal puncture, or reoperation bile accumulation in the abdominal cavity was found during reoperation; 5) Intra-abdominal infection: postoperative abdominal pain, tenderness, rebound tenderness, abdominal muscle tension and other clinical symptoms and signs of peritonitis, increased inflammatory indicators in biochemical examination, abdominal drainage tube drainage fluid or patients with positive bacterial culture in abdominal puncture extract; 6) Digestive tract bleeding: patients with gastrointestinal bleeding symptoms such as hematemesis and melena, and the hemoglobin level is lower than preoperative 20 g/L, or gastrointestinal bleeding is confirmed by endoscopy; 7) Sepsis: clinical symptoms and signs of systemic infection and poisoning such as fear of cold and fever after operation, increased inflammatory indicators in biochemical examination, and positive blood bacterial culture; 8) The patient died.

### 2.7 Economy evaluation

The total cost was defined as the summarization of all fees for the treatment of CBDS. Surgery fee includes surgery fee, anesthesia fee, etc. Treatment cost include the fees for dressing, air pressure therapy, catheterization, gastrointestinal decompression, drainage tube, arteriovenous puncture, intramuscular injection, etc. Medical examination fees include the fees for imaging examination, cardiopulmonary function examination, endoscopy and other related examination. Nursing fees include the fees for indwelling needle nursing, grade nursing and other related nursing. Sanitary materials fees include the fees for catheter, gastric tube, laparoscopic Trocar, dressing drainage tube, and indwelling needle infusion set.

### 2.8 Statistical analysis

All the data analysis were performed by using SPSS 22.0 software. The categorical data or proportions were analyzed by Chi-square test. For the continuous data, the results were presented as mean ± standard deviation, and the significant difference between different groups was analyzed using unpaired t-test. *p* < 0.05 was considered statistically significant.

## 3 Results

### 3.1 Baseline clinical parameters of the patients from endoscopic retrograde cholangiopancreatography group and laparoscopic transcystic common bile duct exploration group

In this study, a total of 89 patients were included in the analysis. In the ERCP group, a total of 26 male and 20 female patients were included; in the LTCBDE group, a total of 18 male and 25 female patients were included. The baseline clinical parameters were shown in Table 1. There was no significant difference in age, bile duct diameter, size of bile stones, number of bile stones, history of abdominal surgy and underlying disease between ERCP group and LTCBDE group (*p* > 0.05; Table 1).

**TABLE 1:** Baseline clinical parameters of patients from ERCP group and LTCBDE group.

Clinical parameters	ERCP group	LTCBDE group	*p* value
Gender			
Male	26	18	0.167
Female	20	25	
Age (years old)	60 ± 14	63 ± 14	0.255
Bile duct diameter (cm)	1.52 ± 0.43	1.73 ± 0.60	0.053
Bile stone diameter (cm)	1.11 ± 0.43	1.33 ± 0.60	0.057
Number of bile stones			
Single	28	18	0.073
Multiple	18	25	
History of abdominal surgery			
None	12	13	0.443
1 time	24	25	
>1 time	10	5	
Underlying disease			
No	22	24	0.924
Yes	24	22	

Underlying disease: 34 cases of hypertension, 12 cases of diabetes mellitus.

### 3.2 Preoperative bile stone-associated complications from endoscopic retrograde cholangiopancreatography group and laparoscopic transcystic common bile duct exploration group

The preoperative bile stone-associated complications in ERCP group and LTCBDE group were shown in Table 2. In the ERCP group, a total of 25 patients had bile stones-associated complications, and the number of patients who had cholangitis, jaundice and pancreatitis were 17, 18 and 5, respectively. In the LTCBDE group, a total of 26 patients had bile stones-associated complications, and the number of patients who had cholangitis, jaundice and pancreatitis were 21, 19 and 5, respectively. There was no significant difference in bile stones-associated complications between ERCP and LTCBDE group (*p* > 0.05; Table 2).

**TABLE 2:** Preoperative bile stone-associated complications from ERCP group and LTCBDE group.

	ERCP group	LTCBDE group	*p* value
Presence of bile stones-associated complications			
No	21	17	0.56
Yes	25	26	
Type of bile stone-associated complications			
Cholangitis	17	21	0.91
Jaundice	18	19	
Pancreatitis	5	5	

### 3.3 Comparison of therapeutic efficacy between endoscopic retrograde cholangiopancreatography group and laparoscopic transcystic common bile duct exploration group

The success rates of surgery in the ERCP and LTCBDE group were 95.7% (44/46) and 97.7% (42/43), respectively. Seven out of 46 patients (15.2%) and 2 out of 43 patients (4.5%) had bile stone residue in the ERCP and LTCBDE group, respectively. There was no significant difference in the success rate of surgery and bile stone residue between ERCP and LTCBDE group (Table 3).

**TABLE 3:** Therapeutic efficacy in ERCP group and LTCBDE group.

	ERCP group	LTCBDE group	*p* value
Success rate of surgery	95.7% (44/46)	97.7% (42/43)	1
Bile stone residue	15.2% (7/46)	4.7% (2/43)	0.193

### 3.4 Postoperative complications between endoscopic retrograde cholangiopancreatography group and laparoscopic transcystic common bile duct exploration group

In the ERCP group, 15 out of 46 patients (32.6%) had postoperative complications, and 3 out of 43 patients (7.0%) had postoperative complications in the LTCBDE group. The incidence of postoperative complications in the LTCBDE group was significantly lower than that in the ERCP group (Table 4). Nine out of 46 patients had pancreatitis in the ERCP group; while none patient had pancreatitis in the laparoscopy group (Table 4). The incidence of pancreatitis in the ERCP group was significantly higher than that in the LTCBDE group (Table 4). Nine out of 46 patients in the ERCP group had a biliary infection, and 2 out of 43 patients had a biliary infection in the laparoscopy group (Table 4). None of the patients had bile leakage in the ERCP group; 1 out of 43 patients (2.3%) had bile leakage. Two out 46 patients in the ERCP group had sepsis; one of the patients had sepsis in the LTCBDE group (Table 4). There was no significant difference in biliary infection, bile leakage and sepsis between the ERCP group and the LTCBDE group (Table 4).

**TABLE 4:** Postoperative complications of ERCP group and LTCBDE group.

	ERCP group	LTCBDE group	*p* value
Prescence of complications	32.6% (15/46)	7.0% (3/43)	0.006
Type of complication			
Biliary infection	19.6% (9/46)	4.7% (2/43)	0.07
Pancreatitis	19.6% (9/46)	0% (0/43)	0.003
Bile leakage	0% (0/46)	2.3% (1/43)	0.483
Sepsis	4.3% (2/46)	0% (0/43)	0.495

### 3.5 Comparison of the cost for treating bile stones in the endoscopic retrograde cholangiopancreatography group and laparoscopic transcystic common bile duct exploration group

In terms of surgery cost, the cost was significantly lower in the ERCP group than that in the LTCBDE group (Table 5). For the treatment cost, the cost was significantly lower in the ERCP group than that in the LTCBDE group (Table 5). There was no significant difference in the cost of medical examination, laboratory cost and medicine cost between ERCP group and LTCBDE group (Table 5). For the cost of nursing, the cost in the ERCP group was lower than that in the LTCBDE group (Table 6); while the cost of sanitary materials in the ERCP group was higher than that in the LTCBDE group (Table 5). There was no significant difference in the total cost between the ERCP group and the LTCBDE group (Table 5).

**TABLE 5:** The cost for treating bile stones in ERCP and LTCBDE group.

	ERCP group	LTCBDE group	*p* value
Surgery (CNY)	2977.17 ± 984.00	6444.88 ± 1080.26	<0.001
Treatment (CNY)	1520.61 ± 623.44	2886.20 ± 872.17	<0.001
Medical examination (CNY)	2415.76 ± 1094.99	2131.23 ± 1588.22	0.325
Laboratory tests (CNY)	1679.52 ± 778.41	1838.50 ± 962.36	0.392
Western medicine (CNY)	9149.55 ± 5948.65	11897.63 ± 6218.32	0.036
Nursing (CNY)	586.24 ± 343.49	890.07 ± 369.27	<0.001
Sanitary materials (CNY)	13677.38 ± 3278.81	5748.16 ± 1745.95	<0.001
Total (CNY)	32701.89 ± 8828.51	33730.19 ± 9604.50	0.6

**TABLE 6:** Length of hospital stay of patients from ERCP group and laparoscopy group.

	ERCP group	LTCBDE group	*p* value
Total length of hospital stay (d)	10.76 ± 4.82	13.70 ± 4.30	0.003
Length of hospital stay before surgery (d)	5.61 ± 3.26	7.51 ± 3.78	0.013
Duration of surgery (min)	62.22 ± 30.30	88.81 ± 39.85	0.001
Length of hospital stay after surgery (d)	5.15 ± 3.16	6.19 ± 2.04	0.072

### 3.6 Length of hospital stay of patients from endoscopic retrograde cholangiopancreatography group and laparoscopic transcystic common bile duct exploration group

The total length of hospital stay in ERCP group and LTCBDE group was 10.76 ± 4.82 days and 13.70 ± 4.30 days, respectively, and the difference was statistically significant (Table 6). In addition, the length of hospital stay before surgery and the duration of surgery in the ERCP were significantly shorter than that in the LTCBDE group (Table 6). On the other hand, there was no significant difference in the length of hospital stay after surgery between ERCP group and LTCBDE group (Table 6).

### 3.7 The cost-effectiveness analysis between endoscopic retrograde cholangiopancreatography group and laparoscopic transcystic common bile duct exploration group

The cost-effectiveness analysis between ERCP and LTCBDE group was shown in Table 7. The cost-effectiveness ratio of ERCP group was 34171.25, and the cost-effectiveness of LTCBDE group was 34524.25 (Table 7). The incremental cost-effectiveness ratio (ICER) of the two groups was 51415 (Table 7).

**TABLE 7:** The cost-effectiveness analysis between ERCP group and laparoscopy group.

	ERCP group	Laparoscopy group
Averaged total cost (CNY)	32701.89	33730.19
Effectiveness	95.70%	97.70%
Cost/Effectiveness ratio	34171.25	34524.25
ICER (∆C/∆E)	--	51415

## 4 Discussion

In this study, the success rate of ERCP was consistent with previous studies reporting that the success rate of ERCP in the treatment of CBDS was about 80–98% (Berggren et al., 1996; Poulose et al., 2007). The surgical approach success rate of common duct stones was in line with the 94–98% reported in relevant literature (Dasari et al., 2013). There was no significant difference in the success of procedure between the two groups. In the ERCP group, there were 2 cases of surgical failures, which was consistent with the 3–10% reported in the literature (Ding et al., 2014).

In this study, there were 7 patients with the residual disease of CBDS in the ERCP group, and 2 patients in the LCTBDE group, which were higher than the residual rate of choledocholithiasis (2.2-3.0%) in the LCTBDE reported in the relevant literature (Dorman and Franklin, 1997). For patients with residual choledocholithiasis after choledocholithiasis, clinicians should choose an appropriate treatment plan according to the patient’s specific condition and their conditions. For patients with T-tube placement, stone removal through the T-tube sinus can be performed again, and duodenoscopy is the first choice for other patients (Anwar et al., 2004). The efficacy of ERCP group and LCTBDE group in the treatment of choledocholithiasis is similar. Clinicians should choose an appropriate treatment plan for patients with choledocholithiasis according to the specific condition of the patient and their conditions.

In this study, neither the ERCP group nor the LTCBDE group had any damage to the abdominal organs and tissues during the operation and postoperative complications. The total postoperative complications in the ERCP group was higher than the 6.3–11.0% reported in the previous study (Park et al., 2014). In this study, postoperative complications were relatively high in the ERCP group, which was partly due to the lack of experience of clinicians in the early stage of this technology in our hospital.

In terms of biliary tract infection, there were 9 cases of postoperative biliary tract infection in the ERCP group, which was 2.4–10.3% higher than the incidence of acute postoperative cholangitis after EST in previous studies (Lu et al., 2014; Li et al., 2018), and 2 cases of postoperative biliary tract infection in the LCTBDE group. In this study, postoperative biliary tract infection mainly occurred in the ERCP group and manifested as abdominal pain, fever, and jaundice in some patients. All of them improved after treatment such as liver protection and anti-infection. The reason was considered to be caused by damage to the duodenal papilla. Damage to the duodenal papilla can lead to edema of the duodenal papilla, leading to poor bile drainage and increasing the chance of biliary tract infection; on the other hand, EST can damage the physiological function of the papillary sphincter, cause intestinal fluid reflux, and increase the chance of retrograde biliary tract infection (Bergman et al., 1997; Sgouros and Pereira, 2006). Therefore, during ERCP, clinicians should minimize the stimulation of the duodenal papilla, preserve the function of the sphincter of Oddi, and place nasobiliary drainage or biliary stent drainage if necessary to help prevent biliary tract infection. The patient’s condition was treated with antibiotics in the perioperative period, thereby reducing the occurrence of biliary tract infection.

In terms of pancreatitis, the number of postoperative pancreatitis cases in the ERCP group was higher than the reported incidence of postoperative pancreatitis after ERCP in the relevant literature, which was about 1.0–15.7% (Choudhary et al., 2011). All patients with postoperative pancreatitis in this study were mild pancreatitis, which was cured by fasting, enzyme inhibition, acid inhibition, liver protection, and anti-infection. The incidence of postoperative pancreatitis in the ERCP group was significantly higher than that in the LTCBDE group. The reason may be that EST destroys the physiological function of the sphincter of Oddi, which easily leads to intestinal reflux and poor pancreatic drainage (Masci et al., 2003). Postoperative nasobiliary drainage, drug prophylaxis, and active postoperative treatment can effectively prevent or reduce the occurrence of pancreatitis after ERCP. The incidence of postoperative complications after ERCP is closely related to the operator’s proficiency. Repeated intubation, pancreatic duct injection and imaging times, and bile duct intubation and imaging success rates are all major risk factors for postoperative pancreatitis (Choudhary et al., 2011). Therefore, this study believes that the prevention of pancreatitis after ERCP includes: 1) completion by physicians with rich clinical experience, 2) avoiding repeated intubation to damage the duodenal papilla, 3) strictly controlling the duodenal papilla Incision and balloon dilatation, 4) strictly grasp the indications and timing of surgery 5) pay attention to the application of contrast agents during the operation 6) actively prevent and treat.

In this study, postoperative bile leakage was mainly manifested as follows: postoperative abdominal drainage tube continued to drain a large amount of bile, localized peritonitis in the right upper quadrant, and limited right upper quadrant effusion was found by abdominal B-ultrasound, and bile was confirmed by the puncture. Bile leakage was successfully controlled after 2 weeks of treatment, including enzyme inhibition, anti-infection, nutritional support, and continuous abdominal drainage. This bile leakage case is a patient with an indwelling T-tube during laparoscopic surgery. The cause of bile leakage is considered to be the bile leakage next to the T-tube, which may be related to the patient’s physical condition and the poor suture of the T-tube by the operator.

In this study, the main clinical manifestations of postoperative sepsis after ERCP were systemic infection symptoms such as chills, chills, and fever after operation, increased inflammatory indicators related to biochemical tests, positive blood bacterial culture, and received anti-infection, fluid replacement, nutritional support, etc. The condition improved after treatment. The occurrence of sepsis in this study may be caused by retrograde infection of the biliary tract after ERCP.

The postoperative complications in the ERCP group and the LCTBDE group were all early complications, and they were all discharged after conservative treatment. In terms of safety, laparoscopic choledocholithiasis is superior to ERCP lithotripsy for the treatment of choledocholithiasis. Although in this study, laparoscopic common bile duct exploration for CBDS was superior to ERCP stone removal in terms of safety, the risk of general anaesthesia and abdominal tissue trauma was avoided in the ERCP group. Some studies have reported that the use of endoscopy shortens the operation time, avoids the greater trauma of abdominal incision and tissue, and reduces the risk of anaesthesia and damage. These minimally invasive advantages are more beneficial to the recovery of patients’ physiological and immune functions.

Hospitalization expenses, length of hospital stay and operation time are clinical economic indicators of a technical level evaluated by the criteria of evidence-based medicine and are also key indicators of hospital management, department performance and medical quality evaluation. There is a wide range of factors that affect the hospitalization cost, length of stay, and operation time of patients, not only related to the disease itself, including the type of disease, degree of disease, chronic underlying disease, etc., but also related to hospital-related management factors, including reducing preoperative examinations, laboratory tests, etc. time, improving the level of medical technology, etc. Reducing medical expenses, shortening hospital stay and operation time will help to improve the utilization rate of medical resources and the level of hospital performance management. The cost of treatment, and sanitary materials was significantly higher in the ERCP group than that in LTCBDE group, which may be related to the LCTBDE group requiring general anaesthesia during the operation, postoperative medication, postoperative dressing change, and postoperative abdominal drainage tube nursing care, etc. The examination fee in the ERCP group was higher than that in the LTCBDE group, which may be related to the use of digestive endoscopy and other examinations in the ERCP group in this study. The cost of sanitary materials in the ERCP group was higher than that in the LCTBDE group, which may be related to the use of guidewire, balloon and biliary stents in some patients in the ERCP group. The average total hospital stay, average preoperative hospital stay and average operative time were longer in the LTCBDE group than in the ERCP group. The reason that the preoperative hospital stay in the LTCBDE group was longer than that in the ERCP group may be related to the need to comprehensively evaluate the patients’ cardiopulmonary function, anaesthesia risk and other tolerance tests before LCTBDE.

Cost-effectiveness analysis is currently the most commonly used economic evaluation method in the field of health care. It determines the most efficient use of resources by analysing the effect obtained after cost consumption. The research scope takes into account both cost and effect, to reflect the economic idea of maximizing economic and social benefits with limited health resources. In this study, in terms of cost-effectiveness ratio (C/E), the ERCP group cost 34,171.25 CNY per unit of treatment effect, and the LCTBDE group cost 34,524.25 CNY per unit of treatment effect. The LCTBDE group had more treatment effects per unit than the ERCP group. From the incremental cost-effectiveness ratio (ΔC/ΔE), the LCTBDE group spent 51415 CNY more than the ERCP group for each additional unit of treatment effect. The results showed that the ERCP group had a significant cost-effectiveness advantage over the LCTBDE group.

In this study, combined with related diagnosis and treatment costs, related hospitalization time, operation time, and cost-effectiveness analysis, the ERCP approach for the treatment of choledocholithiasis has the advantages of comprehensive economic indicators over LCTBDE. However, in the clinical setting, medical workers and patients cannot blindly pursue their economic benefits. On the one hand, the indications and contraindications of ERCP approach should be strictly grasped; on the other hand, ERCP requires expensive medical equipment and superb endoscopic techniques.

There are the following limitations: 1) The sample size is relatively small; 2) It is a single-centre and retrospective study; 3) The observation and follow-up time is short, and some long-term complications (>12 months) of the two minimally invasive procedures are not clear; 4) The ERCP in our hospital is in the early stage, and the clinicians are relatively inexperienced, and there may be bias in the choice of ERCP or LCTBDE for the treatment of patients with CBDS; 5) This study only compared the efficacy, safety and economics of ERCP and LCTBDE in the treatment of CBDS, while choledocholithiasis combined with cholecystolithiasis was not examined.

## 5 Conclusion

In conclusion, ERCP and LTCBDE approaches had similar therapeutic efficacy in the treatment of CBDS. The safety of LTCBDE approach is superior to that of ERCP approach for the treatment of CBDS. ERCP approach is more economical in the treatment of CBDS than LTCBDE approach. Medical workers should be based on the specific conditions of patients with CBDS, combined with their own conditions and technology, to choose a reasonable treatment plan to achieve the best therapeutic effect.

## Data Availability

The original contributions presented in the study are included in the article/supplementary material, further inquiries can be directed to the corresponding author.

## References

[B1] Anwar S. Rahim R. Agwunobi A. Bancewicz J. (2004). The role of ERCP in management of retained bile duct stones after laparoscopic cholecystectomy. N. Z. Med. J. 117 (1203), U1102. 15477926

[B2] Baiu I. Hawn M. T. (2018). Choledocholithiasis. Jama 320 (14), 1506. 10.1001/jama.2018.11812 30304429

[B3] Berggren U. Zethraeus N. Arvidsson D. Haglund U. Jonsson B. (1996). A cost-minimization analysis of laparoscopic cholecystectomy versus open cholecystectomy. Am. J. Surg. 172 (4), 305–310. 10.1016/s0002-9610(96)00197-3 8873518

[B4] Bergman J. J. van Berkel A. M. Groen A. K. Schoeman M. N. Offerhaus J. Tytgat G. N. (1997). Biliary manometry, bacterial characteristics, bile composition, and histologic changes fifteen to seventeen years after endoscopic sphincterotomy. Gastrointest. Endosc. 45 (5), 400–405. 10.1016/s0016-5107(97)70151-2 9165322

[B5] Choudhary A. Bechtold M. L. Arif M. Szary N. M. Puli S. R. Othman M. O. (2011). Pancreatic stents for prophylaxis against post-ERCP pancreatitis: A meta-analysis and systematic review. Gastrointest. Endosc. 73 (2), 275–282. 10.1016/j.gie.2010.10.039 21295641

[B6] Cianci P. Restini E. (2021). Management of cholelithiasis with choledocholithiasis: Endoscopic and surgical approaches. Wjg 27 (28), 4536–4554. 10.3748/wjg.v27.i28.4536 34366622 PMC8326257

[B7] Dasari B. V. Tan C. J. Gurusamy K. S. Martin D. J. Kirk G. McKie L. (2013). Surgical versus endoscopic treatment of bile duct stones. Cochrane Database Syst. Rev. 2013 (12), Cd003327. 10.1002/14651858.CD003327.pub4 PMC6464772 24338858

[B8] Ding G. Cai W. Qin M. (2014). Single-stage vs. two-stage management for concomitant gallstones and common bile duct stones: A prospective randomized trial with long-term follow-up. J. Gastrointest. Surg. 18 (5), 947–951. 10.1007/s11605-014-2467-7 24493296

[B9] Dorman J. P. Franklin M. E. (1997). Laparoscopic common bile duct exploration by choledochotomy. Surg. Innov. 4 (1), 34–41. 10.1177/155335069700400106 10401136

[B10] Friedman G. D. (1993). Natural history of asymptomatic and symptomatic gallstones. Am. J. Surg. 165 (4), 399–404. 10.1016/s0002-9610(05)80930-4 8480871

[B11] Gracie W. A. Ransohoff D. F. (1982). The natural history of silent gallstones. N. Engl. J. Med. 307 (13), 798–800. 10.1056/nejm198209233071305 7110244

[B12] Guo T. Wang L. Xie P. Zhang Z. Huang X. Yu Y. (2021). Surgical methods of treatment for cholecystolithiasis combined with choledocholithiasis: Six years' experience of a single institution. Surg. Endosc. 36 (7), 4903–4911. 10.1007/s00464-021-08843-x 34731303 PMC9160127

[B13] Kawai K. Akasaka Y. Murakami K. Tada M. Kohli Y. Nakajima M. (1974). Endoscopic sphincterotomy of the ampulla of Vater. Gastrointest. Endosc. 20 (4), 148–151. 10.1016/s0016-5107(74)73914-1 4825160

[B14] Kenny R. Richardson J. McGlone E. R. Reddy M. Khan O. A. (2014). Laparoscopic common bile duct exploration versus pre or post-operative ERCP for common bile duct stones in patients undergoing cholecystectomy: Is there any difference? Int. J. Surg. 12 (9), 989–993. 10.1016/j.ijsu.2014.06.013 24998206

[B15] Li T. Wen J. Bie L. Gong B. (2018). Comparison of the long-term outcomes of endoscopic papillary large balloon dilation alone versus endoscopic sphincterotomy for removal of bile duct stones. Gastroenterology Res. Pract. 2018, 1–8. 10.1155/2018/6430701 PMC6051268 30057600

[B16] Lu Y. Wu J. C. Liu L. Bie L. K. Gong B. (2014). Short-term and long-term outcomes after endoscopic sphincterotomy versus endoscopic papillary balloon dilation for bile duct stones. Eur. J. gastroenterology hepatology 26 (12), 1367–1373. 10.1097/meg.0000000000000218 25264985

[B17] Masci E. Mariani A. Curioni S. Testoni P. A. (2003). Risk factors for pancreatitis following endoscopic retrograde cholangiopancreatography: A meta-analysis. Endoscopy 35 (10), 830–834. 10.1055/s-2003-42614 14551860

[B18] Ong T. Z. Khor J. L. Selamat D. S. Yeoh K. G. Ho K. Y. (2005). Complications of endoscopic retrograde cholangiography in the post-MRCP era: A tertiary center experience. World J. Gastroenterol. 11 (33), 5209–5212. 10.3748/wjg.v11.i33.5209 16127754 PMC4320397

[B19] Park J. Y. Jeon T. J. Hwang M. W. Sinn D. H. Oh T. H. Shin W. C. (2014). Comparison between ulinastatin and nafamostat for prevention of post-endoscopic retrograde cholangiopancreatography complications: A prospective, randomized trial. Pancreatology 14 (4), 263–267. 10.1016/j.pan.2014.03.022 25062874

[B20] Petelin J. B. (2003). Laparoscopic common bile duct exploration. Surg. Endosc. 17 (11), 1705–1715. 10.1007/s00464-002-8917-4 12958681

[B21] Poulose B. K. Speroff T. Holzman M. D. (2007). Optimizing choledocholithiasis management. Arch. Surg. 142 (1), 43–48. 10.1001/archsurg.142.1.43 17224499

[B22] Sanchez A. Rodriguez O. Bellorín O. Sánchez R. Benítez G. (2010). Laparoscopic common bile duct exploration in patients with gallstones and choledocholithiasis. Jsls 14 (2), 246–250. 10.4293/108680810x12785289144395 20932377 PMC3043576

[B23] Sgouros S. N. Pereira S. P. (2006). Systematic review: Sphincter of Oddi dysfunction - non-invasive diagnostic methods and long-term outcome after endoscopic sphincterotomy. Aliment. Pharmacol. Ther. 24 (2), 237–246. 10.1111/j.1365-2036.2006.02971.x 16842450

[B24] Shaffer E. A. (2006). Gallstone disease: Epidemiology of gallbladder stone disease. Best Pract. Res. Clin. gastroenterology 20 (6), 981–996. 10.1016/j.bpg.2006.05.004 17127183

[B25] Soltan H. M. Kow L. Toouli J. (2001). A simple scoring system for predicting bile duct stones in patients with cholelithiasis. J. Gastrointest. Surg. official J. Soc. Surg. Alimentary Tract 5 (4), 434–437. 10.1016/s1091-255x(01)80073-1 11985986

[B26] Vilallonga R. Fort J. M. Iordache N. Armengol M. Clèries X. Solà M. (2012). Use of images in a surgery consultation. Will it improve the communication? Chir. Buchar. Rom. 107 (2), 213–217. 22712351

[B27] Williams E. Beckingham I. El Sayed G. Gurusamy K. Sturgess R. Webster G. (2017). Updated guideline on the management of common bile duct stones (CBDS). Gut 66 (5), 765–782. 10.1136/gutjnl-2016-312317 28122906

[B28] Williams E. J. Green J. Beckingham I. Parks R. Martin D. Lombard M. (2008). Guidelines on the management of common bile duct stones (CBDS). Gut 57 (7), 1004–1021. 10.1136/gut.2007.121657 18321943

